# Ultrafine Pd Nanoparticles Supported on Soft Nitriding Porous Carbon for Hydrogen Production from Hydrolytic Dehydrogenation of Dimethyl Amine-Borane

**DOI:** 10.3390/nano10081612

**Published:** 2020-08-17

**Authors:** Zhaoyu Wen, Qiong Fu, Jie Wu, Guangyin Fan

**Affiliations:** College of Chemistry and Materials Science, Sichuan Normal University, Chengdu 610068, China; zhaoyuwen10@163.com (Z.W.); libre1614@163.com (Q.F.); fanguangyin@sicnu.edu.cn (G.F.)

**Keywords:** nano-catalyst, dimethyl amine-borane, palladium nanoparticles, hydrolysis, soft nitriding

## Abstract

Simple and efficient synthesis of a nano-catalyst with an excellent catalytic property for hydrogen generation from hydrolysis of dimethyl amine-borane (DMAB) is a missing piece. Herein, effective and recycled palladium (Pd) nanoparticles (NPs) supported on soft nitriding porous carbon (NPC) are fabricated and applied for DMAB hydrolysis. It is discovered that the soft nitriding via a low-temperature urea-pretreatment induces abundant nitrogen-containing species on the NPC support, thus promoting the affinity of the Pd precursor and hindering the agglomeration of formed Pd NPs onto the NPC surface during the preparation process. Surface-clean Pd NPs with a diameter of sub-2.0 nm deposited on the NPC support (Pd/NPC) exhibit an outstanding catalytic performance with a turnover frequency (TOF) of 2758 h^−1^ toward DMAB hydrolysis, better than many previous reported Pd-based catalysts. It should be emphasized that the Pd/NPC also possesses a good stability without an obvious decrease in catalytic activity for DMAB hydrolysis in five successive recycling runs. This study provides a facile but efficient way for preparing high-performance Pd catalysts for catalytic hydrogen productions.

## 1. Introduction

Although traditional fossil fuels can provide massive energies for versatile uses in modern society, their large-scale consumptions arouse serious energy crises and environmental pollutions. In view of sustainable development, hydrogen is regarded as an alternative to fossil fuels because of its high energy density and clean burning nature [1,2,3]. However, the safe storage and transportation of hydrogen still hinders its large-scale applications. To mitigate this issue, solid hydrogen storage materials have been demonstrated as a safe and efficient strategy to meet the urgent requirement in hydrogen utilizations [4,5,6,7,8]. Thereinto, dimethyl amine-borane (DMAB) has been regarded as one of the most investigated hydrogen storage materials for hydrogen production [9]. To accelerate fast reaction kinetics, an effective catalyst is required to trigger hydrogen liberation from DMAB hydrolysis.

Heterogeneous catalysts have received intensive attention due to their benefits in catalyst separation and recycling [10,11,12,13,14,15]. Thereinto, Pd has sparked increasing research interest because of its high efficiency toward DMAB dehydrogenation [16], while its high price and limited supply restrain the large-scale applications. The development of Pd-based catalysts with a low loading and high catalytic efficiency is needed. Previous studies have shown that catalytic efficiencies are largely dependent on the sizes of noble metal particles [17]. However, current ways of loading metal on catalyst supports through co-precipitation and impregnation lack size control to some extent. To overcome this limitation, large amounts of protecting molecules are applied to control the size of metal NPs during the synthetic process [18,19]. The massive uses of such protecting molecules also arouse the decline of catalytic activity because of the unavoidable coverage of catalytically active sites [20]. In addition, the removal of such protecting molecules under harsh conditions also results in the loss of catalytic activity [21]. Recent studies have indicated that the heteroatom-doped carbon materials can be used as ideal platforms to synthesize ligand-free metal NPs for catalytic applications [17,22,23,24]. However, most of the synthetic strategies for heteroatom-doping of carbon matrixes are conducted by strong acid treatment and high temperature calcination, leading to the production of carbon materials with relatively low content of heteroatom, which are adverse to the preparation of metal NPs with ultrafine sizes [25].

Inspired by the above consideration, we report a simple, high-efficiency and potentially scalable strategy to synthesize ultrafine Pd NPs with a diameter of sub-2.0 nm firmly bonded on the NPC support prepared by a low temperature calcination of porous carbon with the nitrogen-rich and readily available chemical of urea. It is found that the soft nitriding of PC support induces an abundance of surface nitrogen-containing groups, which are beneficial for the affinity of Pd precursor/Pd NPs on the support and thus inhibit the overgrowth of Pd NPs during the synthetic process. The as-prepared Pd/NPC displays a high turnover frequency (TOF) of 2758 h^−1^ toward DMAB hydrolytic dehydrogenation, which is higher than that of many previously reported Pd-based catalysts. Additionally, the developed catalyst is stable and can be recycled at least five times without obvious deactivation for DMAB hydrolysis. This study supplies a facile and effective way to synthesize a high-performance Pd-based catalyst toward hydrogen generation from DMAB hydrolysis.

## 2. Materials and Methods

### 2.1. Materials

Potassium palladium (II) chloride (K_2_PdCl_4_) was obtained from Kunming Boren Precious Metals Co., Ltd. (Kunming, China). Urea and ethanol were supplied by Aladdin Industrial Corp. (Shanghai, China). Porous carbon (PC) was provided by GuangXi University (Guangxi, China). Sodium borohydride (NaBH_4_) was bought form Alfa Aesar (Shanghai, China). Dimethyl amine-borane (DMAB) was purchased from Sigma-Aldrich (St. Louis, MO, USA).

### 2.2. Synthesis of NPC

NPC was prepared through a gentle thermal decomposition of urea in the presence of PC. Specifically, 0.75 g of urea and 0.5 g of PC were thoroughly grinded in a mortar. Afterward, the mixture was sealed in a crucible, and then annealed at 150 °C for 2 h and 300 °C for another 2 h. The acquired product was washed with water and ethanol for three times. Finally, the as-synthesized NPC was dried at 60 °C overnight for further use.

### 2.3. Preparation of Pd/NPC

In a typical procedure, 100 mg of NPC was dispersed into 200 mL of water and sonicated for 30 min. Then, 200 μL of K_2_PdCl_4_ aqueous solution (10 mg/mL) was added to the suspension and stirred for 2 h. After that, 1 mL of NaBH_4_ solution (1 mg/mL) was rapidly injected into the suspension and followed by stirring for another 2 h to reduce the Pd ions. The collected solid was washed with water and ethanol for several times and dried under vacuum at room temperature for 24 h. The Pd loading of Pd/NPC was 1.0 wt% determined by inductively coupled plasma optical emission spectrometry (ICP-OES). For comparison, Pd/NPC samples with different Pd loadings were prepared under identical conditions except for the introduction of different content of Pd precursor solution.

### 2.4. Characterization

Transmission electron microscopy (TEM) was conducted on JEM-2100F (JEOL, Akishima City, Japan). X-ray diffraction (XRD) patterns of the samples were recorded on a Regaku D/Max-2500 diffractometer (Tokyo, Japan) in the range of 2θ = 5–90°. X-ray Photoelectron Spectroscopy (XPS) was performed on a Thermo ESCALAB 250 Axis Ultra spectrometer (Waltham, MA, USA). Pd loadings in Pd/NPC samples and the leaching test during recycling were determined by ICP-OES with an SPECTRO ARCOS spectrometer (SPECTRO, Kleve, Germany).

### 2.5. Catalytic Procedure

DMAB hydrolysis catalyzed by Pd/NPC was carried out as follows. In a typical procedure, 13 mg of Pd/NPC was mixed with 4 mL of water at room temperature. The mixture was treated by ultrasonication to get a uniform suspension. Afterward, the desired amount of DMAB dissolved in 1 mL of water was injected. The volume of hydrogen generation was recorded by water-displacement method. The kinetic studies for DMAB hydrolysis catalyzed by Pd/NPC were performed at different conditions (catalysts mass, Pd loading, DMAB concentration and reaction temperature).

## 3. Results and Discussion

**Scheme 1:**
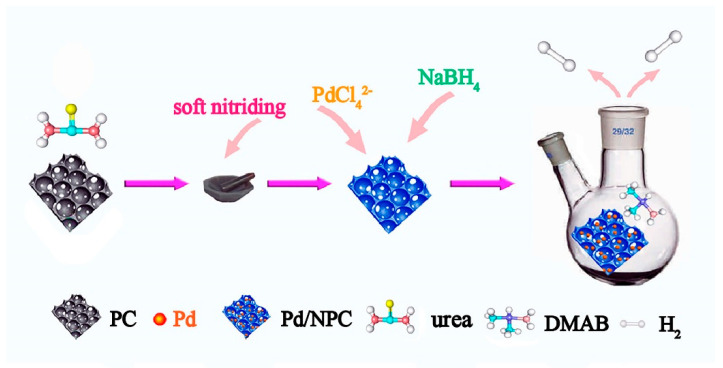
The illustration of the synthetic route of Pd/NPC.

Scheme 1 shows the specifically synthetic procedure for Pd/NPC. First, the obtained PC was homogenously mixed with urea via a mortar grinding. Then, the mixture was put into a muffle furnace and calcined for 2 h at 150 °C and then for another 2 h at 300 °C. Afterward, the cleaned and dried NPC was used as a NP support for the fabrication of Pd/NPC via the adsorption of Pd ions in aqueous solution, followed by a reduction processing with NaBH_4_. The obtained Pd/NPC was applied as a catalyst for DMAB hydrolysis.

**Figure 1:**
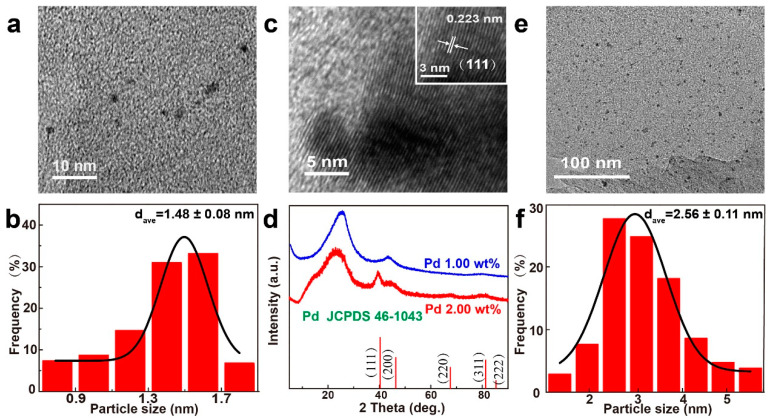
(**a**) TEM image, (**b**) Pd size distribution, (**c**) HRTEM image, and (**d**) XRD patterns of Pd/NPC with different loadings. (**e**) TEM image and (**f**) size distribution of Pd/NPC after recycling test.

The structure and morphology of Pd/NPC were firstly analyzed by TEM. As shown in Figure 1a,b, the metal NPs with an average size of 1.48 nm are uniformly dispersed on the NPC surface without obvious aggregation. The high-resolution TEM (HRTEM) image in Figure 1c confirms the lattice fringes of Pd (111) plane with an interplanar distance of 0.223 nm, verifying that well-crystallized Pd nanocrystals are successfully loaded on the NPC surface. The element mapping images in Figure S1 reveal that the foreign elements of N, O, and Pd are evenly distributed on the support. The XRD pattern of Pd/NPC with a Pd loading of 1.00 wt% exhibits invisible Pd characteristic peaks because of the ultrafine and/or low loading of Pd. This can be verified by the XRD pattern of Pd/NPC with an increasing Pd loading of 2.00 wt%. As shown in Figure 1d, the XRD pattern of Pd/NPC with a higher Pd loading shows obvious diffraction peaks at 40.11°,46.66°,68.12°,82.10°, and 86.62°, corresponding to the (100), (111), (200), (220), (311), and (222) crystal planes of Pd nanocrystal. The surface-clean, ultrafine and homogenous dispersed Pd NPs in Pd/NPC with Pd loading of 1.00 wt% are expected to supply substantial large number of active sites for DMAB hydrolysis to produce hydrogen.

**Figure 2:**
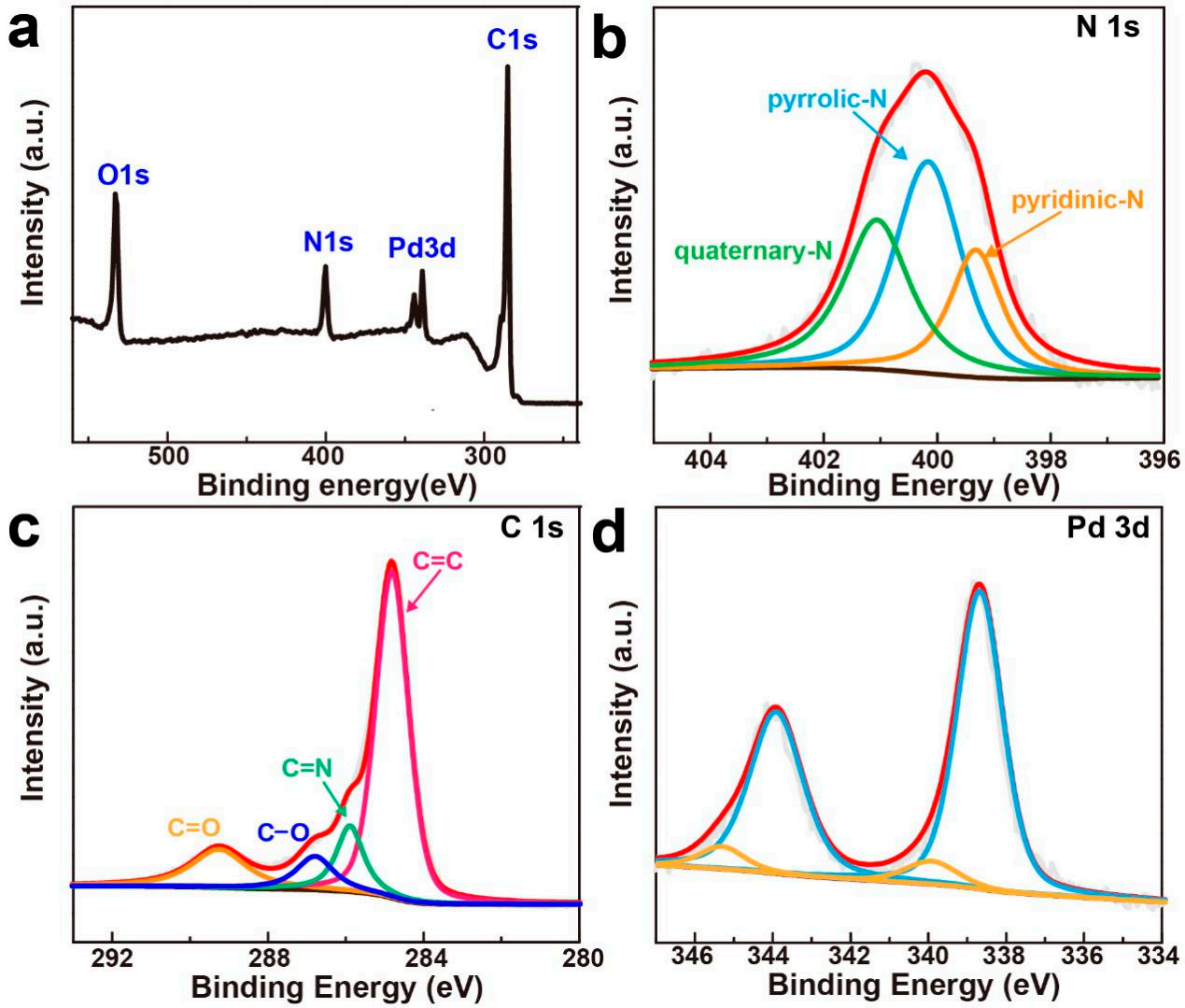
(**a**) The XPS survey spectrum and high-resolution XPS spectra of (**b**) N 1s, (**c**) C 1s, and (**d**) Pd 3d of Pd/NPC.

An XPS survey scan result clearly gives the information that the Pd/NPC is composed of C, Pd, N, and O, suggesting that Pd NPs are successfully introduced to the NPC support (Figure 2a) [22]. The surface atomic percentages of C, Pd, N and O were 71.21, 1.30, 10.98, and 16.53%, respectively. High-resolution of N1s spectrum can be divided into three peaks at 399.1, 400.1, and 401.0 eV, corresponding to the pyridinic-N, pyrrolic-N, and quaternary-N (Figure 2b) [26]. Previous studies have suggested that N-doping can activate nearby carbon atoms, and thus increase the active sites [27,28]. For the C1s spectrum (Figure 2c), the main peak at 284.8 eV is related to the C=C bonds [29,30], and the other two peaks at higher binding energies of 286.5 and 289.3 eV are ascribed to the oxygen-containing groups of C–O and O–C=O, respectively [31,32]. Additionally, the peak located at 285.4 eV is ascribed to C=N [33,34], suggesting the successful nitrogen-doping of PC via the soft nitriding processing [35].The high-resolution Pd 3d core level spectrum (Figure 2d) can be divided into two groups peaks corresponding to oxidized Pd species as a result of the oxidation occurred on the surface of the catalyst during the preparation and characterization procedures.

**Figure 3:**
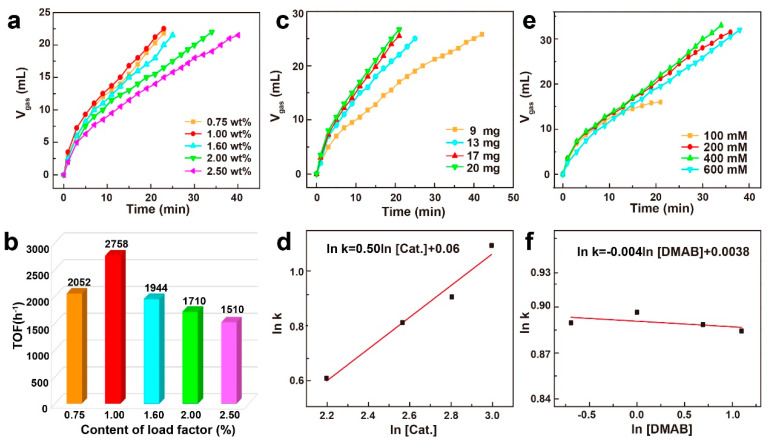
(**a**) Hydrogen production vs time over Pd/NPC with varied Pd loadings. (**b**) The corresponding TOF values. (**c**) Hydrogen generation vs time over Pd/NPC with varied catalysts mass. (**d**) Plot of ln rate vs. ln [Cat.]. (**e**) Hydrogen evolution vs. time over Pd/NPC with varied DMAB concentrations. (**f**) Plot of ln rate vs. ln [DMAB].

The catalytic activity of Pd/NPC was investigated for DMAB hydrolysis. To gain more kinetic information, DMAB hydrolysis on Pd/NPC was performed under different reaction conditions with varied Pd loading and DMAB concentrations. The Pd/NPC catalysts with different Pd loadings of 0.75, 1.00, 1.60, 2.00, and 2.50 wt% were investigated for DMAB hydrolysis at 25.0 °C and the results are shown in Figure 3a. It can be seen that the DMAB hydrolysis reaction was immediately started without any induction period with the generation of one equivalent of hydrogen. The TOF values of Pd/NPC samples gradually increase with the increase of Pd loadings. A maximum TOF value of 2758 h^−1^ is acquired over the Pd/NPC catalyst with a Pd loading of 1.00 wt%. This value is superior to most of the results reported previously (Table 1). Further increasing the Pd loading leads to a gradual decrease of TOF value (Figure 3b). Accordingly, the Pd/NPC with a Pd loading of 1.00 wt% was explored to study other factors affecting the catalytic performance toward DMAB decomposition.

To study the effect of catalyst concentration on DMAB hydrolysis, plots of hydrogen production vs time over Pd/NPC with 1.00 wt% Pd loading at various Pd concentrations were constructed in Figure 3c. The results indicate that the highest catalytic activity for DMAB hydrolysis is observed with the addition of 13 mg Pd/NPC catalyst. The line slope of the plot of hydrogen generation rate vs catalyst mass in a logarithmic scale is 0.50, which indicates that the catalyst mass plays a positive effect on the decomposition of DMAB with Pd/NPC (Figure 3d).

The influence of substrate concentration on DMAB hydrolysis was researched by changing the concentration of DMAB from 100 to 600 mM while keeping other conditions constant. As shown in Figure 3e, the reaction rate for DMAB over Pd/NPC is almost independent of DMAB concentration. The plot of hydrogen generation rate vs DMAB concentration, both in logarithmic scales, was constructed in Figure 3f. A slope of −0.04 of the fitting line is observed, illustrating that DMAB hydrolysis with Pd/NPC can be regarded as a zero-order reaction with DMAB concentration [40].

**Figure 4:**
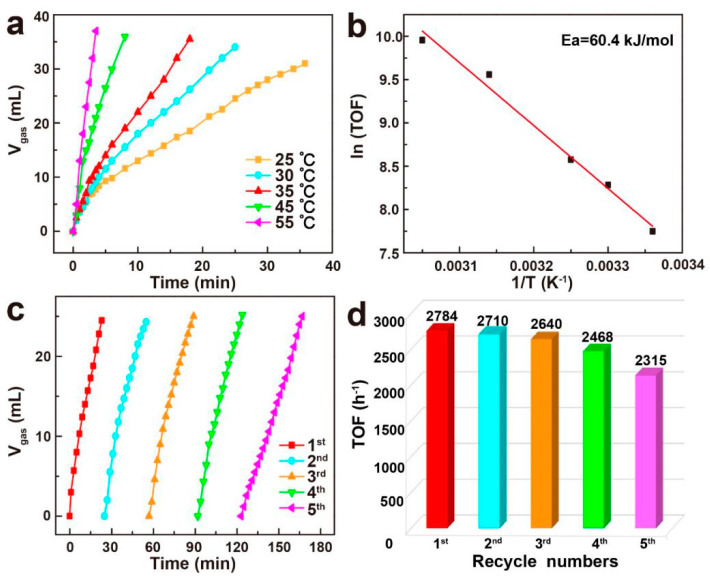
(**a**) Plots of hydrogen generation vs time for DMAB hydrolysis over Pd/NPC at different temperatures. (**b**) Arrhenius plot. (**c**) Time dependence of hydrogen evolution vs time over Pd/NPC for reusability testing for DMAB hydrolysis. (**d**) Corresponding TOF values.

To investigate the effect of reaction temperature on DMAB hydrolysis, a series of control experiments was carried out at different temperatures ranging from 25 to 55 °C while maintaining the other conditions unchanged. The hydrogen generation rate for DMAB hydrolysis rapidly rises with increasing temperature (Figure 4a). The reaction temperature has an enormous influence on catalytic activity of Pd/NPC for DMAB hydrolysis. The activation energy can be calculated based on the slope from the Arrhenius plot. Figure 4b shows the Arrhenius plot of ln (TOF) vs 1/T, from which the *Ea* value is calculated to be 60.4 kJ/mol.

Consequently, the excellent catalytic property of Pd/NPC for DMAB hydrolysis is analyzed accordingly. (1) The clean, ultrasmall and highly distributed sub-2.0 nm Pd NPs on the surface of NPC as determined by TEM analysis can supply an abundance of catalytic surface-active sites for DMAB hydrolysis. (2) The NPC substrate can efficiently host an abundance of Pd NPs, facilitate the mass transfer of DMAB to diffuse in the internal surface of the catalyst [41]. As a result, more DMAB molecules are efficiently reacted and lead to a high catalytic activity. (3) The soft nitriding enables the introduction of pyridinic nitrogen sites which serve as nitrogen ligands for chemical bonding, whereas the oxygen-containing groups supply surface charges for electrostatic adsorption, which may help to anchor Pd atoms and impede the overgrowth of Pd NPs on the NPC surface without the use of extra surface capping agents [42,43]. Thus, the achieved surface-clean and ultrasmall size of Pd/NPC enable a large number of their constituent atoms to be exposed readily available surface reactive sites [44]. Attributing to the above analysis, the Pd/NPC possesses an excellent catalytic performance for hydrogen evolution from DMAB hydrolysis.

Under the optimal reaction conditions, the reusability of Pd/NPC was studied for DMAB hydrolysis. The Pd/NPC can be recycled at least five times (Figure 4c) and only a slight increase in reaction time is observed, indicating that this catalyst has good reusability for DMAB hydrolysis. Specifically, the TOF value of Pd/NPC still reaches 2315 h^−1^ in the fifth run (Figure 4d). The leaching tests by ICP showed that only a negligible Pd leaching was determined during the recycling. Thus, the morphology and size of Pd/NPC after recycling were further detected by TEM. As shown in Figure 1e, TEM image of Pd/NPC after reusability testing indicates a slight aggregation of Pd NPs with the increased size from 1.48 to 2.56 nm (Figure 1f). As a result, the slight decrease of catalytic activity of Pd/NPC during reusability testing should be ascribed to the aggregation of Pd NPs [45]. More studies should be performed to improve the recyclability of the Pd/NPC catalyst toward DMAB hydrolysis.

## 4. Conclusions

In summary, a convenient and efficient method was developed to synthesize ultrafine Pd NPs supported on the NPC surface. The high dispersed and surface-clean Pd NPs with an average particle size of 1.48 nm were produced and showed an excellent catalytic performance toward DMAB hydrolysis at room temperature. A very high TOF value of 2758 h^−1^ was obtained under the optimal conditions, which was higher than that of many of the reported results. Meanwhile, the Pd/NPC also exhibited good reusability for DMAB hydrolysis with five successive runs. Pd/NPC can be considered as a potential nano-catalyst with features of excellent catalytic properties, a simple synthetic method, and good reusability for hydrogen production from DMAB hydrolysis.

## Figures and Tables

**Table 1:** Comparison results of Pd catalysts for DMAB hydrolysis.

Catalysts	*E*_a_ (kJ /mol)	Turnover Frequency (TOF) (mL_H2_ h^−1^g^−1^_metal_)	References
PdNi@GO	38 ± 2	271.9	[35]
RuNi@PVP	36.52 ± 3	330	[36]
PdNi@PEDOT	50.78 ± 2	451.3	[37]
Pd@GO	16.5	38.0	[38]
RuCo@f-MWCNT	13.72 ±2	775.3	[39]
PdNPs@Cu(btc)_2_	173.5 ± 16.2	75	[16]
PdIr@AC	36.6 ± 2	295.1	[40]
Pd@AC	Not mentioned	100	[40]
Pd/NPC	60.4	2758	This work

## Supplementary Materials

The following are available online at https://www.mdpi.com/2079-4991/10/8/1612/s1, Figure S1: SEM images and mapping images of Pd/NPC.
