# Supplementary material for: Ultrafine Pd Nanoparticles Supported on Soft Nitriding Porous Carbon for Hydrogen Production from Hydrolytic Dehydrogenation of Dimethyl Amine-Borane
Source: Nanomaterials (Basel). 2020 Aug 17;10(8):1612. doi: 10.3390/nano10081612 (PMC7466676; doi:10.3390/nano10081612)
Supplement: Supplementary file 1 [file nanomaterials-10-01612-s001.pdf]

nanomaterials

Supplementary Materials

Ultrafine Pd Nanoparticles Supported on Soft Nitriding Porous Carbon for Hydrogen Production from Hydrolytic Dehydrogenation of Dimethyl Amine-Borane

Zhaoyu Wen, Qiong Fu, Jie Wu * and Guangyin Fan

College of Chemistry and Materials Science, Sichuan Normal University, Chengdu 610068, China; zhaoyuwen10@163.com (Z.W.); libre1614@163.com (Q.F.); fanguangyin@sicnu.edu.cn (G.F.)

* Correspondence: wujie@sicnu.edu.cn; Tel.: +86-28-8476-0802

Materials

Potassium palladium (II) chloride (K_2PdCl_4) was obtained from Kunming Boren Precious Metals Co., Ltd, China. Urea and ethanol were supplied by Aladdin Industrial Corp., China. Porous carbon (PC) was provided by GuangXi University, China. Sodium borohydride ($NaBH_4$) was bought form Alfa Aesar. Dimethyl amine-borane (DMAB) was purchased from Sigma-Aldrich.

Characterization

Transmission electron microscopy (TEM) was conducted on JEM-2100F (JEOL). X-ray diffraction (XRD) patterns of the samples were recorded on a Regaku D/Max-2500 diffractometer in the range of $2\theta = 5–90^{\circ}$. X-ray Photoelectron Spectroscopy (XPS) was performed on a Thermo ESCALAB 250 Axis Ultra spectrometer. Pd loadings in Pd/NPC samples and the leaching test during recycling were determined by inductively coupled plasma optical emission spectrometry (ICP-OES) with an Opima 8000 equipment.

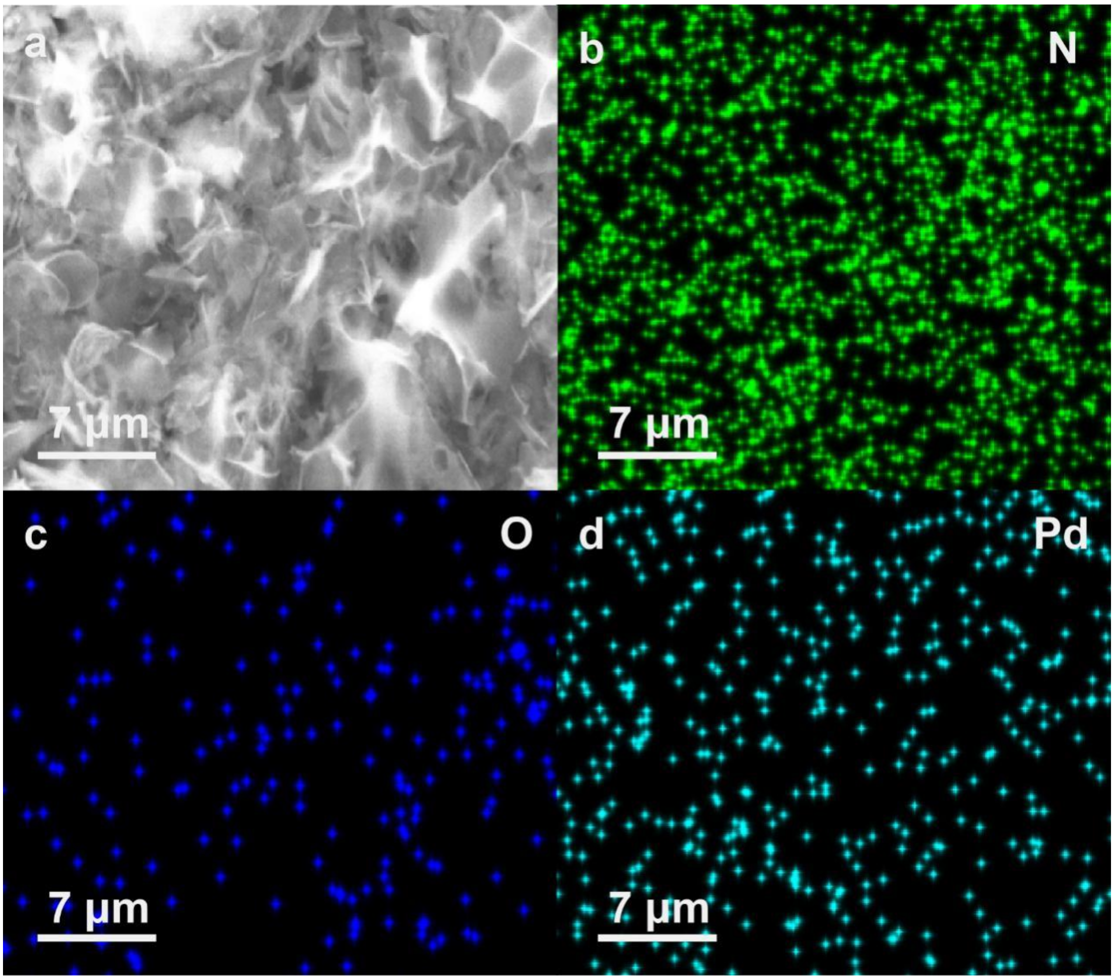

Figure S1. (a) SEM images and mapping images of **(b)** N, **(c)** O and **(d)** Pd for Pd/NPC.
